# Beyond *Fumigatus*: a molecular portrait of clinical *Aspergillus* diversity, pathogenicity, and antifungal resistance

**DOI:** 10.1128/aac.01184-25

**Published:** 2026-01-13

**Authors:** Chioma I. Aneke, Sherin S. Shahegh, Alexandra F. Freeman, Christa Zerbe, Kyung J. Kwon-Chung, Steven Holland, Kevin Fennelly, Michail S. Lionakis, Amir Seyedmousavi

**Affiliations:** 1 Microbiology Service, Department of Laboratory Medicine, Clinical Center, National Institutes of Health 2511, Bethesda, Maryland, USA; 2 Laboratory of Clinical Immunology and Microbiology, National Institute of Allergy and Infectious Diseases, National Institutes of Health 2511, Bethesda, Maryland, USA; 3 Pulmonary Clinical Medicine Section, Cardiovascular Pulmonary Branch, Division of Intramural Research, National Heart, Lung, and Blood Institute, National Institutes of Health 2511, Bethesda, Maryland, USA; University of Iowa, Iowa City, Iowa, USA

**Keywords:** aspergillosis, clinical *Aspergillus *diversity, non-fumigatus *Aspergillus *species, cryptic *Aspergillus *species, antifungal susceptibility, MALDI-TOF MS, species-level identification, pathogenicity

## Abstract

*Aspergillus* infection poses a major clinical challenge, particularly in immunocompromised individuals, with invasive diseases associated with high mortality. While *Aspergillus fumigatus* remains the predominant species causing human infections, recent studies highlight the growing clinical significance of lesser-known and cryptic *Aspergillus* species, which often exhibit reduced susceptibility to standard antifungal therapies. In this study, we analyzed 196 clinical *Aspergillus* isolates from 107 patients treated at the NIH Clinical Center between 2019 and 2022. A total of 38 *Aspergillus* species across 11 taxonomic sections were identified, with non-*fumigatus* and cryptic species accounting for 77.1% of all isolates. The most frequently recovered species were *A. fumigatus sensu stricto* (22.9%), *A. sydowii* (8.7%), *A. calidoustus* (7.1%), *A. nidulans* (6.6%), *A. tanneri* (6.1%), and *A. terreus* (5.6%). Species-level identification was achieved in 43% of isolates using matrix-assisted laser desorption/ionization time-of-flight mass spectrometry (MALDI-TOF MS). In contrast, PCR sequencing confirmed species identity in over 88% of isolates by targeting the internal transcribed spacer (ITS) region of rDNA, 81% using the β-tubulin gene, and 68% using the calmodulin gene. The most common underlying clinical conditions among patients were bronchiectasis (35%), chronic granulomatous disease (22%), and pulmonary non-tuberculous mycobacterial infection (17%). Out of 107 patients, eight died (8/107, 7.5%); six of these deaths occurred in patients with chronic granulomatous disease (CGD) and two in patients with RAG1 deficiency. Antifungal susceptibility testing showed that olorofim had the lowest minimal inhibitory concentrations across species. In contrast, the activity of triazoles and amphotericin B was variable, particularly against *A. tanneri*, *A. calidoustus*, and *A. sydowii*. This study presents one of the largest species-level data sets of *Aspergillus* isolates to date, underscoring the diversity, pathogenic potential, and resistance profiles of non-*fumigatus* species. Accurate species identification plays an important role in guiding appropriate antifungal therapy and improving clinical outcomes, although further studies are needed to elucidate its direct impact on treatment decisions.

## INTRODUCTION

*Aspergillus* species are a major cause of fungal-related morbidity and mortality worldwide, particularly among individuals with chronic obstructive pulmonary disease (1), patients in intensive care units, and those with underlying conditions such as hematopoietic stem cell transplantation or hematological malignancies (2). An estimated 2.1 million individuals are affected annually, with allergic bronchopulmonary aspergillosis and chronic pulmonary aspergillosis alone accounting for over 1.8 million cases globally (3).

Among species in the genus *Aspergillus*, *A. fumigatus* is the most frequently encountered species in clinical settings and is the leading cause of aspergillosis. It is associated with a broad spectrum of disease manifestations, ranging from allergic reactions and non-invasive colonization to aspergilloma formation and life-threatening invasive disease, particularly in immunocompromised individuals (4, 5). In recognition of its clinical significance and rising antifungal resistance, the World Health Organization (WHO) designated *A. fumigatus* as a critical priority fungal pathogen in 2022. Beyond *A. fumigatus*, several other *Aspergillus* species—including *A. flavus*, *A. terreus, A. nidulans*, *A. versicolor*, *A. sydowii*, *A. niger*, *A. tubingenesis*, and the less common *A. lentulus* and *A. udagawae*—have also been reported in human and animal infections (6, 7). Emerging cryptic species—such as *A. fumisynnematus* (8), *A. lentulus* (9), *A. udagawae* (10), and *A. viridinutans* (6)—which are often morphologically indistinguishable from *A. fumigatus*, pose additional diagnostic and therapeutic challenges due to their frequent resistance to first-line antifungal agents (11, 12).

Conventional mold identification in clinical laboratories primarily relies on culture-based methods, with less frequent use of matrix-assisted laser desorption/ionization time-of-flight mass spectrometry (MALDI-TOF MS) or polymerase chain reaction (PCR) targeting the internal transcribed spacer (ITS) region of ribosomal DNA (rDNA) for sequencing-based identification. However, the accuracy of both MALDI-TOF MS and ITS sequencing has limitations in accurately distinguishing cryptic and non-*fumigatus Aspergillus* species, which may result in delays in appropriate diagnosis and suboptimal treatment (13).

Given the high mortality associated with misdiagnosed or inadequately treated *Aspergillus* infections (14), precise species identification and antifungal susceptibility profiling are essential for effective clinical management. In this study, we aimed to investigate the species diversity and *in vitro* antifungal susceptibility patterns of *Aspergillus* isolates obtained from patients hospitalized at the National Institutes of Health (NIH) Clinical Center.

## MATERIALS AND METHODS

### Fungal isolates

A total of 196 clinical *Aspergillus* isolates were collected between 1 January 2019 and 31 December 2022, from patients treated at the NIH Clinical Center—a 200-bed research hospital in Bethesda, Maryland, USA. The center treats patients from a wide range of geographic regions, both within the United States and internationally. *Aspergillus* isolates were obtained from various types of clinical specimens, including respiratory samples (i.e., sputum, bronchoalveolar lavage fluid, tracheal aspirates, bronchial wash, and pleural fluid), tissues from biopsies/autopsies (i.e., lung, liver, bone, sinus), abscesses and other sterile body fluids (i.e., drainage, blood), and skin/nail lesions (Fig. S1). Initial identification was based on macroscopic colony characteristics and microscopic examination of conidia and conidiophore structures, as performed by experienced mycology laboratory personnel. Table S1 provides detailed information on each patient’s underlying condition, source of isolates, species identification, and corresponding GenBank accession numbers.

### MALDI-TOF mass spectrometry identification

Species-level identification was conducted using MALDI-TOF MS with the MALDI Biotyper system (Bruker Daltonics Inc., Billerica, MA, USA). Fungal biomass (approximately the size of a small pencil eraser) was transferred into 70% ethanol and silica beads, then lysed for 30 s at 4,000 rpm using a high-speed tissue homogenizer (MO BIO PowerLyser, Qiagen, USA). Proteins released into the supernatant were used for analysis. Identification was performed using two databases: Bruker Filamentous Fungi Library 2.0 (FFL2.0) and NIH in-house fungal database. Identification scoring thresholds were applied as follows: ≥2.0: species-level identification; 1.7–1.99: genus-level identification; and <1.7: inconclusive identification.

### DNA extraction, amplification, and sequencing

Isolates were cultured on Sabouraud dextrose agar at 30°C for 2–7 days. Conidia were then harvested for genomic DNA extraction using the DNeasy UltraClean Microbial Kit (Qiagen GmbH, Germany), following the manufacturer’s instructions. DNA purity and concentration were evaluated using a NanoDrop 1000 Spectrophotometer. Species confirmation was performed via PCR amplification and sequencing of the ITS region of rDNA, as well as the β-tubulin (β-tub) and calmodulin (CaM) genes, as previously described (15–18). Primer sequences are listed in Table S2.

### Alignment and phylogenetic reconstruction

The resulting DNA sequences were checked and assembled via SeqMan in the Lasergene package. The newly generated sequences were blasted in the NCBI GenBank and MycoBank databases. Identification was confirmed only if sequence similarity was ≥99%. Sequences were submitted to the GenBank database under the accession numbers provided in Table S1. Phylogenetic reconstructions were performed using maximum likelihood analysis with RAxML (raxmlHPC-PTHREADS-AVX) based on the ITS, β-tub, and CaM gene regions. The best-fit substitution model was selected for each locus, and 1,000 bootstrap replicates were conducted to assess node support. The resulting phylogenetic trees were visualized and annotated using iTOL (Interactive Tree of Life, web-based platform).

### Antifungal susceptibility testing

*In vitro* antifungal susceptibility testing was conducted according to the Clinical and Laboratory Standards Institute (CLSI) M38-A3 guidelines (19). The following antifungal agents were evaluated: amphotericin B (AmB), itraconazole (ITC), voriconazole (VRC), posaconazole (POS), isavuconazole (ISA), terbinafine (TRB), micafungin (MFG), and olorofim (OLF). All antifungal agents, except OLF (F2G, Ltd., UK), were obtained from Sigma-Aldrich (USA). For all agents except micafungin, the minimum inhibitory concentration (MIC) was defined as the lowest drug concentration that completely inhibited visible growth, assessed visually by comparison with a drug-free control. For micafungin, the minimum effective concentration (MEC) was defined as the lowest concentration that produced abnormal, short, and branched hyphal structures, in contrast to the typical long, unbranched hyphae observed in the control well. Details regarding conidial inoculum densities, ranges of drug concentration, and incubation durations for determining MICs and MECs are provided in Table S3. To ensure the reliability and reproducibility of the results, *Candida krusei* (ATCC 6258), *Aspergillus fumigatus* (ATCC MYA-3626), and *Trichophyton mentagrophytes* (MRL1957, ATCC MYA-44394) were used as quality control strains on each testing day. Each experiment was conducted in three independent replicates on different days. The interpretation of VRC and ISA MICs for *A. fumigatus* was based on the CLSI M38M51S-ED3:2022 (20) clinical breakpoints: for VRC, MICs ≤0.5 µg/mL were considered susceptible, 1 µg/mL intermediate, and ≥2 µg/mL resistant; for ISA, MICs of 1 µg/mL were classified as susceptible, 2 µg/mL as intermediate, and ≥4 µg/mL as resistant.

### Data analysis

The geometric mean (GM), range, and MIC/MEC 50/90 distributions were analyzed using GraphPad Prism version 9.0 for Windows (GraphPad Software, San Diego, CA).

## RESULTS

### Patient groups and clinical sources of isolates

A total of 196 *Aspergillus* strains were isolated from clinical specimens obtained from 107 patients (Table S1). The annual distribution of isolates was 26 in 2019, 33 in 2020, 39 in 2021, and 75 in 2022. The highest number of isolates (156/196; 79%) was obtained from respiratory specimens (Fig. S1), including 125 from sputum (63.8%) and 27 from bronchoalveolar lavage samples (13.8%). Additional sources included abscesses and sterile body fluids (19 isolates; 10%), biopsies of lung, liver, and bone (15 isolates; 8%), and skin, nails, and soft tissue (6 isolates; 3%) (Table S4). As shown in Fig. S2, the greatest number of isolates came from patients with bronchiectasis (*n* = 68; 35%). The most frequently identified species in this group were *A. fumigatus sensu stricto* (*n* = 25), followed by *A. calidoustus* (*n* = 4), *A. fumisynnematus* (*n* = 4), *A. flavus* (*n* = 3), *A. lentulus* (*n* = 3), *A. nidulans* (*n* = 3), *A. niger* (*n* = 3), *A. sydowii* (*n* = 3), *A. terreus* (*n* = 3), and others (Table S5 ). The second most prevalent respiratory condition was pulmonary non-tuberculous mycobacterial (NTM) infection, accounting for 17.5% (*n* = 34) of isolates. The predominant species in this group were *A. fumigatus sensu stricto* (*n* = 12), *A. sydowii* (*n* = 9), and *A. terreus* (*n* = 5). Isolates from patients with primary immunodeficiencies and inborn errors of immunity accounted for 32% (*n* = 62), with chronic granulomatous disease (CGD) being the most prevalent diagnosis (43/62; 69.5%). Review of clinical data also revealed that among 107 patients, eight (7.5%) died, including six with CGD and two with RAG1 deficiency (Table S1). The mortality rate among CGD patients due to *Aspergillus* was 60% (6/10), with fatal infections caused by the following non-*fumigatus Aspergillus* species: *A. calidoustus*, *A. felis*, *A. pseudoviridinutans*, *A. tanneri*, and *A. udagawae*. In addition, both patients with RAG1 deficiency, who also died (100% mortality), were infected with *A. calidoustus* and *A. fumigatus sensu stricto*.

### *Aspergillus* diversity

**Fig 1:**
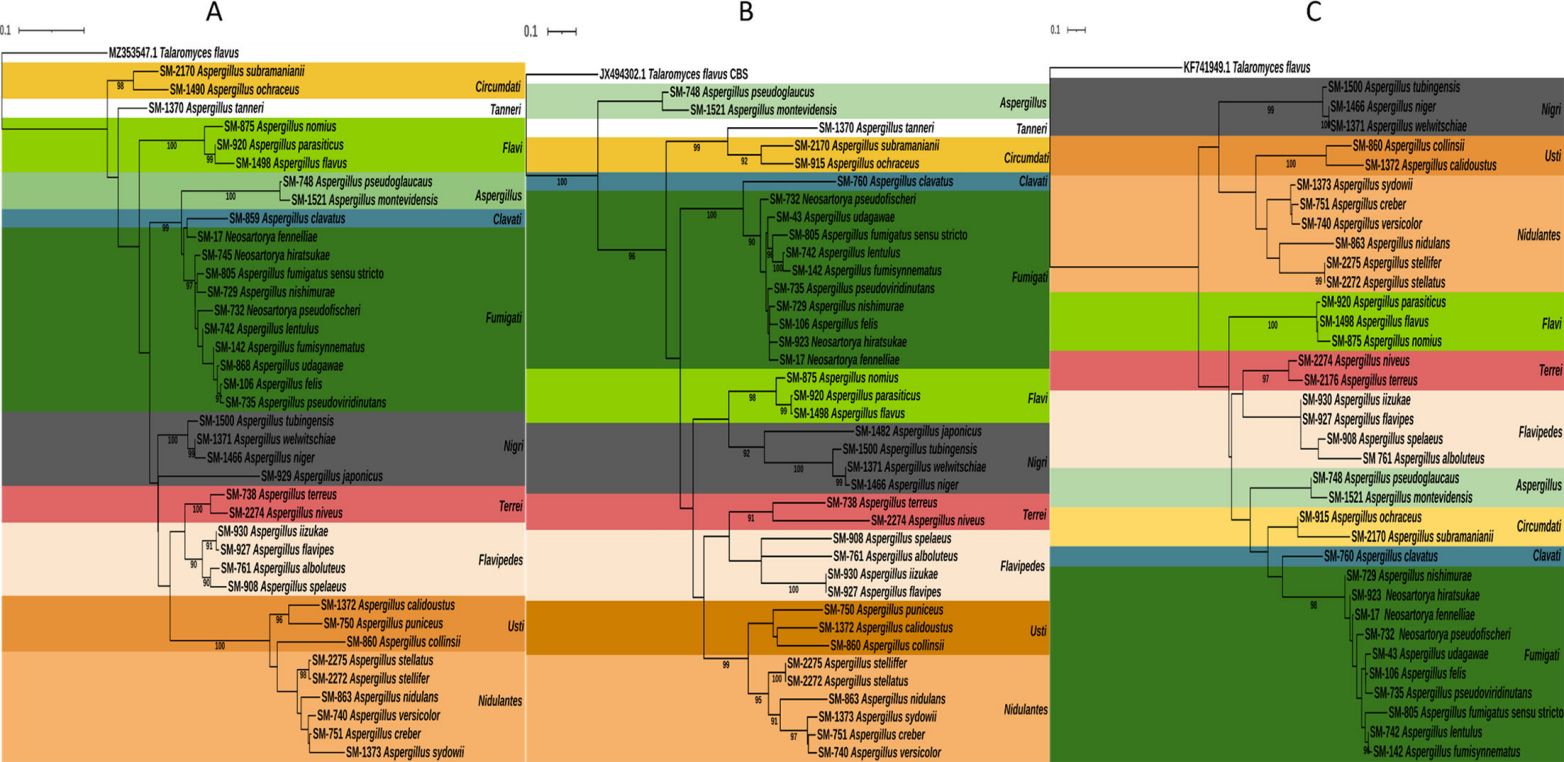
Phylogenetic trees of selected clinically relevant *Aspergillus* species isolated in this study based on ITS (**A**), β-tubulin (**B**), and calmodulin (**C**) gene sequences using maximum likelihood analysis.

**Fig 2:**
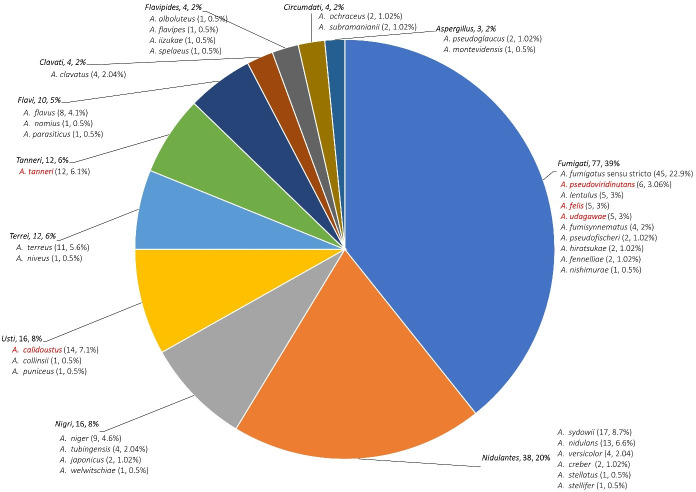
Taxonomic distribution of 196 clinical *Aspergillus* isolates across 11 sections and 38 species.

To determine the evolutionary relationships among *Aspergillus* species, sequences from ITS, β-tub, and the CaM genes were used for phylogenetic analysis (Fig. 1). The maximum likelihood tree constructed from these sequences demonstrated clear phylogenetic separation of clinically important *Aspergillus* species into distinct sections. The 196 clinical *Aspergillus* isolates were taxonomically diverse, representing 38 distinct species across 11 sections (Fig. 2). The most prevalent sections among the isolates were *Fumigati* with 77 isolates (39%), followed by *Nidulantes* with 38 isolates (20%), and *Nigri* and *Usti*, each with 16 isolates (8%). Other notable groups included *Terrei* and *Tanneri* (12 isolates each; 6%), *Flavi* (10 isolates; 5%), *Clavati*, *Flavipedes*, and *Circumdati* (4 isolates each; 2%), and *Aspergillus* with 3 isolates (1.53%).

Within section *Fumigati*, *Aspergillus fumigatus sensu stricto* was the predominant species, representing 58.5% (45/77) of the isolates in this group (Fig. S3A). However, cryptic and non-*fumigatus* species accounted for a significant proportion (41.5%), including *A. pseudoviridinutans* (*n* = 6; 7.8%), *A. lentulus* (*n* = 5; 6.5%), *A. felis* (*n* = 5; 6.4%), *A. udagawae* (*n* = 5; 6.5%), and *A. fumisynnematus* (*n* = 4; 5.2%). Section *Nidulantes* was predominantly represented by *A. sydowii* (17 isolates; 44.7%) and *A. nidulans* (13 isolates; 34.2%) (Fig. S3B). Similarly, section *Usti* was largely dominated by *A. calidoustus* (Fig. S3C), accounting for 87.5% of the isolates (*n* = 14). Notably, while *A. fumigatus sensu stricto* remained the most frequently isolated species (41/156; 26.3%) from lower respiratory tract samples, *A. sydowii* (17/156; 10.9%), and *A. calidoustus* (13/156; 8.3%) also emerged as prominent pathogens, underscoring the clinical significance of these non-*fumigatus* species.

### Identification methods (MALDI-TOF MS vs PCR-sequencing)

MALDI-TOF MS correctly identified 76.5% of *Aspergillus* isolates with a score >1.70, demonstrating the highest accuracy in the sections *Fumigati*, *Nidulantes*, *Nigri*, *Usti*, *Terrei*, and *Flavi* (Table 1). In contrast, identification of species within section *Tanneri* was limited due to its underrepresentation in available MALDI-TOF MS reference libraries. Species-level identification by PCR sequencing was achieved using three genetic targets: ITS (88.3%), β-tub (81.1%), and CaM (68.4%) (Table 1). BLAST analysis of ITS sequences in GenBank confirmed a similar taxonomic distribution, with the most represented sections being *Fumigati* (*n* = 77; 39%), *Nidulantes* (*n* = 38; 20%), *Usti* and *Nigri* (*n* = 16 each; 8%), *Terrei* and *Tanneri* (*n* = 12 each; 6%), *Flavi* (*n* = 10; 5%), followed by *Clavati*, *Flavipedes*, and *Circumdati* (*n* = 4 each; 2%), and *Aspergillus* (*n* = 3; 1.53%). The GenBank accession numbers corresponding to all 196 isolates are provided in Table 1.

**TABLE 1:** Comparison of MALDI-TOF MS and multilocus PCR-sequencing (ITS, β-tubulin, CaM) for species-level identification of *Aspergillus* species

Section	Number of isolates	MALDI score			ITS	Btub	CaM
		<1.70	≥1.70				
			1.70–1.99	≥2.0			
*Fumigati*	78	14	20	44	75	76	56
*Nidulantes*	38	8	15	15	33	20	27
*Nigri*	16	2	3	11	13	12	12
*Usti*	16	4	8	4	8	11	12
*Terrei*	13	3	6	4	13	10	10
*Flavi*	10	0	7	3	10	10	8
*Tanneri*	10	10	0	0	9	10	2
*Clavati*	4	1	2	1	3	3	2
*Flavipides*	4	1	3	0	3	1	2
*Circumdati*	4	2	1	1	4	4	2
*Aspergillus*	3	1	1	1	2	2	1
Total	**196**	**46**	**66**	**84**	**173**	**159**	**134**
			**33.67%**	**42.86%**			
		**23.47%**	**76.53%**		**88.27%**	**81.12**	**68.37**

### Antifungal susceptibility patterns

Antifungal susceptibility testing revealed considerable variation in MIC/MECs (Table 2). The novel antifungal agent OLF and the echinocandin drug MFG demonstrated the lowest GM MICs, with OLF ranging from 0.016 to 0.5 µg/mL and MFG from 0.016 to 1 µg/mL, indicating strong activity against all tested isolates (100%). OLF was particularly effective against isolates in sections *Fumigati, Nidulantes, Terrei*, and *Flavi*, with GM MICs as low as 0.02–0.03 µg/mL. Among the triazoles, POS exhibited the most consistent activity across species, with GM MICs typically between 0.09 and 0.4 µg/mL. However, elevated MICs were observed in section *Clavati* (GM: 3 µg/mL) and section *Usti* (e.g., *A. calidoustus* GM: 14.9 µg/mL), indicating reduced susceptibility. In contrast, ITC, VRC, and ISA displayed greater inter- and intra-species variability. Notably, *A. lentulus, A. felis*, and *A. pseudoviridinutans* from section *Fumigati* exhibited higher MICs to ITC (overall GM MICs: 0.19–13.1 µg/mL), and VRC (overall GM MICs: 0.31–11.8 µg/mL), suggesting reduced susceptibility or resistance (Table S5).

**TABLE 2:** Distribution of the MIC/MEC of eight antifungal agents against *Aspergillus* species isolated from 196 clinical samples as determined by the CLSI M38-A3 method

Section (no. of isolates)	*Aspergillus* sp. (no. of isolates)	Antifungal	Range	GM	MIC/MEC (μg/mL)										
					0.016	0.031	0.062	0.125	0.25	0.5	1	2	4	8	16
*Aspergillus* (2)	*A. pseudoglaucus* (1)	AMB												1	
		ITC									1				
		VOR											1		
		POS							1						
		ISA										1			
		TRB								1					
		MCF			1										
		OLF					1								
	*A. montevidensis* (1)	AMB										1			
		ITC							1						
		VOR									1				
		POS						1							
		ISA									1				
		TRB												1	
		MCF				1									
		OLF				1									
*Circumdati* (4)	*A. ochraceus* (2)	AMB	0.125–16	8				1							1
		ITC	0.062–0.125	0.07			1	1							
		VOR	0.25–0.5	0.4					1	1					
		POS	0.062–0.125	0.09			1	1							
		ISA	0.125–0.5	0.3				1		1					
		TRB	0.016–1	1	1						1				
		MCF		0.031		2									
		OLF	0.031–0.062	0.05		1	1								
	*A. subramanianii* (2)	AMB	2–4	3								1	1		
		ITC	0.25–1	0.62					1		1				
		VOR		0.25					2						
		POS		0.25					2						
		ISA	0.062–0.125	0.09			1	1							
		TRB	0.25–0.5	0.4					1	1					
		MCF	0.062–0.125	0.09			1	1							
		OLF		0.016	2										
*Clavati* (4)	*A.* *clavatus* (4)	AMB	0.016–0.5	0.38	1					3					
		ITC	0.5–16	4.63						1	2				1
		VOR	1–4	2.25							1	2	1		
		POS	1–2	3							2	2			
		ISA	1–2	1.25							3	1			
		TRB	0.125–0.25	0.2				2	2						
		MCF	0.016–0.062	0.03	3		1								
		OLF		0.016	4										
*Flavi* (10)	*A. flavus* (8)	AMB	0.5–4	1.88							5	1	2		
		ITC	0.062–0.5	0.3			1	3		4					
		VOR	0.5–2	0.94						3	4	1			
		POS	0.062–0.25	0.12			2	5	1						
		ISA	0.25–2	0.84					1	3	3	1			
		TRB	0.031–0.25	0.09		1	5	1	1						
		MCF	0.016–0.062	0.03	3	4	1								
		OLF	0.016–0.062	0.03	4	3	1								
	*A. nomius* (1)	AMB										1			
		ITC									1				
		VOR										1			
		POS								1					
		ISA										1			
		TRB							1						
		MCF							1						
		OLF					1								
	*A. parasiticus* (1)	AMB								1					
		ITC						1							
		VOR									1				
		POS						1							
		ISA									1				
		TRB			1										
		MCF				1									
		OLF			1										
*Flavipides* (4)	*A. alboluteus* (1)	AMB										1			
		ITC							1						
		VOR								1					
		POS						1							
		ISA									1				
		TRB							1						
		MCF			1										
		OLF				1									
	*A. flavipes* (1)	AMB								1					
		ITC						1							
		VOR									1				
		POS					1								
		ISA											1		
		TRB						1							
		MCF				1									
		OLF			1										
	*A. iizukae* (1)	AMB								1					
		ITC						1							
		VOR									1				
		POS					1								
		ISA											1		
		TRB						1							
		MCF			1										
		OLF			1										
	*A. spelaeus* (1)	AMB										1			
		ITC							1						
		VOR							1						
		POS						1							
		ISA						1							
		TRB								1					
		MCF									1				
		OLF				1									
*Fumigati* (76)	*A. fumigatus sensu stricto* (45)	AMB	0.125–16	1.22				3	11	8	15	6	1		1
		ITC	0.062–16	2.06			1	9	15	14		1			5
		VOR	0.25–8	1.55					3	13	22	3	1	4	
		POS	0.016–1	0.15	1	7	16	14	3	4	1				
		ISA	0.25–8	1.59					2	13	22	4	1	4	
		TRB	0.062–16	4.79			1			1	2	21	10	5	6
		MCF	0.016–0.125	0.03	32	9	3	2							
		OLF	0.016–0.125	0.03	17	23	6	1							
	*A. pseudoviridinutans* (6)	AMB	0.5–4	2.3						1	1	2	2		
		ITC	0.5–16	3.6						1		1			1
		VOR	1–4	2.2							1	4			
		POS	0.125–1	0.4				1	3	1	1				
		ISA	1–4	1.7							4	1	1		
		TRB	0.25–0.5	0.3					5	1					
		MCF		0.02	6										
		OLF	0.016–0.062	0.03	4	1									
	*A. lentulus* (5)	AMB	1–16	9.8							1			2	2
		ITC	0.125–16	6.6			1			2					2
		VOR	0.5–4	3.3					1	1			4		
		POS	0.031–0.5	0.2		1		2	1	1					
		ISA	0.25–2	1.7					1			4			
		TRB	0.062–1	0.5			1		2		2				
		MCF	0.016–0.031	0.02	4	1									
		OLF	0.016–0.031	0.02	4										
	*A. felis* (5)	AMB	0.5–4	2						1	2	1	1		
		ITC	1–16	4.6							3		1		1
		VOR	2–4	3.6								1	4		
		POS	0.125–0.5	0.4				1	1	3					
		ISA	1–8	3.8							1	1	2	1	
		TRB	0.125–2	0.7				1	2		1	1			
		MCF		0.016	5										
		OLF	0.016–0.062	0.03	2	2									
	*A. udagawae* (4)	AMB	0.5–2	1.1						1	2	1			
		ITC	0.062–0.25	0.2			2	1		1					
		VOR	0.5–1	0.8		1				2	2				
		POS	0.031–0.25	0.1		1	2		1						
		ISA	0.5–1	0.9						1	3				
		TRB	0.125–1	0.5				1		2	1				
		MCF	0.016–0.031	0.02	3	1									
		OLF	0.016–0.031	0.02	3	1									
	*A.* *fumisynnematus* (4)	AMB	1–4	2.8							1	1	2		
		ITC	0.062–0.25	0.2			1	1	2						
		VOR	0.25–0.5	0.4					1	3					
		POS	0.031–0.125	0.09		1	1	2							
		ISA	0.25–0.5	0.4					1	3					
		TRB	0.125–0.5	0.3				1	2	1					
		MCF		0.016	4										
		OLF		0.016	4										
	*N. fennelliae* (2)	AMB		1							2				
		ITC	0.5–1	0.8						1					
		VOR		1							2				
		POS	0.125–0.5	0.3				1		1					
		ISA		1							2				
		TRB	0.062–0.125	0.09			1	1							
		MCF	0.016–0.031	0.02		2									
		OLF		0.016	2										
	*N. hiratsukae* (2, 2.6)	AMB	2–4	3								1	1		
		ITC	0.5–1	0.8						1	1				
		VOR	1–2	1.5							1	1			
		POS		0.25					2						
		ISA	0.5–1	0.8						1	1				
		TRB	0.062–0.125	0.09			1	1							
		MCF		0.016	2										
		OLF		0.016	2										
	*A. nishimurae* (1)	AMB								1					
		ITC						1							
		VOR								1					
		POS					1								
		ISA									1				
		TRB							1						
		MCF			1										
		OLF			1										
	*N. pseudofischeri* (2)	AMB		1							2				
		ITC		1							2				
		VOR		2								2			
		POS	0.25–0.5	0.4					1	1					
		ISA	1–2	1.5							1	1			
		TRB	0.25–0.5	0.4					1	1					
		MCF		0.016	2										
		OLF		0.016	2										
*Nidulantes* (37)	*A. sydowii* (17)	AMB	0.5–16	2.5						2	7	5	2		1
		ITC	0.125–4	0.8				2	2	8	3	1	1		
		VOR	0.25–4	1.5					1	2	6	7	1		
		POS	0.125–1	0.3				3	10	3	1				
		ISA	0.25–4	1.1					1	2	12	1	1		
		TRB	0.062–4	0.5			3	10	2			1	1		
		MCF	0.016–0.062	0.03	8	6	3								
		OLF	0.016–0.031	0.02	15	2									
	*A. nidulans* (13)	AMB	0.25–2	1					3	2	5	3			
		ITC	0.062–2	0.6			1	2	4	2	3	1			
		VOR	0.125–1	0.6				1	3	5	4				
		POS	0.031–0.5	0.2		1	3	2	4	3					
		ISA	0.062–0.5	0.2			1	6	3	3					
		TRB	0.125–2	0.5				3	4	4	1	1			
		MCF	0.016–0.25	0.05	7	4		1	1						
		OLF	0.016–0.062	0.04	1	7	5								
	*A. versicolor* (3)	AMB	0.5–2	1.2						1	1	1			
		ITC	0.25–0.5	0.4					1	2					
		VOR	0.5–1	0.7						2	1				
		POS	0.125–0.25	0.2				1	2						
		ISA	0.5–1	0.8						1	2				
		TRB	0.25–0.5	0.4					1	2					
		MCF	0.016–0.062	0.04	1	1	1								
		OLF		0.016	3										
	*A. creber* (2)	AMB	1.5	1–2							1	1			
		ITC	0.25–0.4						1	1					
		VOR	0.5–1	0.8						1	1				
		POS		0.125				2							
		ISA	0.125–1	0.6				1			1				
		TRB	0.25–1	0.6					1		1				
		MCF	0.016–0.031	0.02	1	1									
		OLF	0.016–0.031	0.02	1	1									
	*A. stellatus* (1)	AMB										1			
		ITC										1			
		VOR								1					
		POS									1				
		ISA							1						
		TRB									1				
		MCF				1									
		OLF					1								
	*A. stellifer* (1)	AMB										1			
		ITC								1				N	
		VOR								1					
		POS						1							
		ISA												1	
		TRB							1						
		MCF			1										
		OLF				1									
*Nigri* (16)	*A. niger* (9)	AMB	0.25–2	1					1	3		5			
		ITC	0.25–16	2					2	2	4				1
		VOR	0.5–2	1						2	4	3			
		POS	0.125–0.5	0.27				2	5	2					
		ISA	1–4	2							2	6	1		
		TRB	0.062–0.25	0.118			3	5	1						
		MCF		0.016	9										
		OLF	0.031–0.062	0.06		2	6	1							
															
	*A. tubingensis* (4)	AMB	0.125–1	0.53				1`		2	1				
		ITC	0.5–1	0.75						2	2				
		VOR	1–2	1.5							2	2			
		POS	0.125–0.5	0.28				1	2	1					
		ISA	2–4	2.5								3	1		
		TRB	0.125–0.25	0.19				2	2						
		MCF		0.016	4										
		OLF		0.062			4								
	*A. japonicus* (2)	AMB	0.25–0.5	0.37					1	1					
		ITC	0.016–0.062	0.039	1		1								
		VOR	0.062–0.25	0.16			1		1						
		POS	0.016–0.125	0.07	1			1							
		ISA	0.062–0.125	0.09			1	1							
		TRB	0.062–0.125	0.09			1	1							
		MCF	0.016–0.062	0.04	1		1								
		OLF		0.016	2										
	*A. welwitschiae* (1)	AMB									1				
		ITC									1				
		VOR							1						
		POS						1							
		ISA									1				
		TRB								1					
		MCF		1											
		OLF				1									
*Tanneri* (9)	*A. Tanneri* (9)	AMB	0.031–16	7.6		1				2		2			4
		ITC	0.25–2	0.64					3	4	1	1			
		VOR	0.031–2	0.89		1				2	5	1			
		POS	0.031–0.5	0.18		1	3	3		2					
		ISA	1–4	1.44							7	1	1		
		TRB	0.031–0.125	0.08		4	1	4							
		MCF	0.016–0.5	0.15	5	1			1	2					
		OLF	0.031–0.25	0.09		3	3	2	1						
*Terrei* (12)	*A. terreus* (11)	AMB	0.5–4	2.59						1	2	3	5		
		ITC	0.031–1	0.4		1	2		1	6	1				
		VOR	0.25–2	1.14					2	2	3	4			
		POS	0.031–0.5	0.17		2	1	4	3	1					
		ISA	0.25–4	1.7					1	3	3	1	3		
		TRB	0.062–1	0.35			1	2	4	3	1				
		MCF	0.016–0.062	0.03	8		3								
		OLF	0.016–0.031	0.02	7	4									
	*A. niveus* (1)	AMB										1			
		ITC								1					
		VOR											1		
		POS							1						
		ISA												1	
		TRB								1					
		MCF				1									
		OLF				1									
*Usti* (16)	*A. calidoustus* (14)	AMB	1.7	0.5–4						1	7	4			
		ITC	0.5–4	1.7						1	7	4			
		VOR	0.125–16	13.9				1				1			12
		POS	0.25–16	14.9				1							13
		ISA	0.5–16	4.5							1	1	11		1
		TRB	0.125–2	0.8				2	4	3	2	3			
		MCF	0.016–0.25	0.016	7	2	1	2	2						
		OLF	0.031–0.5	0.3		1			8	5					
	*A. collinsii* (1)	AMB										1			
		ITC						1							
		VOR							1						
		POS						1							
		ISA								1					
		TRB					1								
		MCF			1										
		OLF			1										
	*A. puniceus* (1)	AMB											1		
		ITC													1
		VOR													1
		POS													1
		ISA												1	
		TRB								1					
		MCF						1							
		OLF							1						

AmB showed variable activity both within and between species. GM MICs ranged from 0.38 µg/mL (*A. clavatus*) to as high as 7.7 µg/mL (*A. tenneri*). Notably, isolates from section *Usti,* especially *A. calidoustus* and *A. puniceus*, had high AmB MICs (GM: 1.8 µg/mL and MICs up to 4 µg/mL), consistent with intrinsic resistance patterns.

TRB exhibited significant activity across all sections except *Fumigati* (MICs: 0.062–16 µg/mL), with the highest MIC values noted in *A. fumigatus sensu stricto*, *A. lentulus*, and members of section *Aspergillus* (GM MIC: 4.25 µg/mL). Isolates from section *Usti*—notably *A. calidoustus* and *A. puniceus*—consistently showed elevated MICs to multiple antifungal classes, including AmB (0.5–4 µg/mL), ITC, VRC, POS, and ISA (range MIC: 0.125–16 µg/mL), highlighting their multidrug-resistant nature.

Emerging species such as *A. tanneri* displayed a broad range of MICs, particularly for AmB (0.031–16 µg/mL) and ISA (1–4 µg/mL), indicating variable susceptibility. Interestingly, *A. collinsii*, another isolate from section *Usti*, showed uniformly low MICs to all tested antifungals except AmB (MIC: 2 µg/mL), suggesting it may represent a more susceptible species among the members of this section (Table 2).

## DISCUSSION

This study provides an in-depth characterization of species diversity, genetic profiles, and antifungal susceptibility of *Aspergillus* isolates recovered from clinical specimens of patients with diverse underlying conditions—including primary immunodeficiencies that confer heightened susceptibility to severe fungal infections. To our knowledge, this is one of the most extensive investigations in recent years focusing not only on the prevalence of *A. fumigatus sensu stricto* but also on cryptic and emerging *Aspergillus* species across 11 taxonomic sections.

The predominance of respiratory tract specimens (79%) as the source of isolates is consistent with previous reports, which identified the lungs as the primary portal of *Aspergillus* entry and colonization in immunocompromised hosts (21, 22). Our findings highlight the remarkable diversity of *Aspergillus* species infecting patients with primary immunodeficiencies (PIDs), revealing distinct associations between specific fungal species and particular immunodeficiency syndromes. Invasive aspergillosis remains a major clinical challenge in individuals with inborn errors of immunity. In our cohort, patients with CGD experienced a 60% mortality rate despite antifungal therapy and demonstrated a unique species distribution profile. Notably, CGD patients were found to be susceptible to *Aspergillus* species that are less commonly encountered in otherwise healthy individuals, including *A. tanneri*, *A. pseudoviridinutans*, *A. felis*, *A. udagawae*, and *A. calidoustus*, which can be associated with aggressive clinical courses and intrinsic resistance to conventional antifungal agents. These organisms are increasingly linked to breakthrough infections and antifungal treatment failures (6, 9). Our data underscores the urgent need for early diagnosis, species-level fungal identification, and tailored treatment strategies, particularly in high-risk populations. Accurate species identification is critical, as antifungal susceptibility can vary substantially among *Aspergillus* species (9, 23). Moreover, specific PID subtypes appear to predispose patients to specific *Aspergillus* profiles (24, 25), likely reflecting the nature of the underlying immune defect. NADPH oxidase deficiency in CGD compromises neutrophil function, rendering patients vulnerable to uncommon *Aspergillus* species not typically seen in other immunodeficiencies (4). Several studies have reported differences in phagocytosis kinetics among *Aspergillu*s species. For instance, *A. nidulans* is phagocytosed more slowly and induces a lower oxidative burst with fewer reactive oxygen intermediates compared to *A. fumigatus*, which may help explain the higher incidence of *A. nidulans* infections in patients with CGD (26, 27). In contrast, patients with antibody deficiencies or impaired mucosal defenses—such as those with bronchiectasis—more frequently harbor *A. fumigatus*, *A. terreus*, and related species. In bronchiectasis and NTM patients, *Aspergillus* detection likely represents a combination of colonization and opportunistic infection in structurally damaged airways (28).

In total, we identified 38 distinct *Aspergillus* species spanning 11 sections and a wide range of underlying conditions, emphasizing the rising clinical relevance of rare and cryptic species. Section *Fumigati* was the most prevalent (39%), with *A. fumigatus sensu stricto* remaining the dominant species. This aligns with previous reports from Australia, Brazil, China, New Zealand, and the USA, where *A. fumigatus* is consistently the most commonly isolated species in immunocompromised populations, including patients with cystic fibrosis (CF) or non-CF bronchiectasis (22, 29–33). However, 61% of isolates in our cohort were non-*fumigatus Aspergillus* species, including *A. calidoustus*, *A. felis*, *A. lentulus*, *A. pseudoviridinutans*, *A. sydowii*, and *A. udagawae*. These emerging pathogens are increasingly recognized in clinical contexts, especially among patients with hematologic malignancies and PIDs. Notably, sections *Nidulantes* and *Usti*, traditionally considered primarily environmental, accounted for 19% and 8% of isolates, respectively, underscoring their growing clinical importance.

The frequent recovery of *A. sydowii* and *A. calidoustus* from lower respiratory tract samples further supports their pathogenic potential, particularly in individuals with chronic lung diseases or underlying immunosuppression (34–36). Our findings differ from earlier studies that predominantly identified *A. fumigatus*, *A. flavus*, *A. terreus,* and *A. niger* as the most common species from the lower respiratory tract (31, 32). These discrepancies may reflect differences in environmental exposure, patient populations, and technical advances in the detection of cryptic species.

MALDI-TOF MS is increasingly implemented in clinical microbiology laboratories for fungal identification, particularly due to its speed and reliability in identifying yeasts. Its application to mold diagnostics is also progressing, though challenges remain (37). In this study, MALDI-TOF MS achieved an overall identification accuracy of 76.5%, with higher accuracy for section *Fumigati* (44/78, 56.4%) and section *Nigri* (11/16, 68.8%), while showing notably lower identification rates in section *Nidulantes* (15/38, 39.5%), *Usti* (4/16, 25%), *Terrei* (4/13, 30.8%), and *Flavi* (3/10, 30%). Notably, *A. tanneri* was not identified by MALDI-TOF MS, likely reflecting the absence of this species in current reference databases. These findings reinforce the need for expanded, curated spectral libraries—especially for rare and cryptic *Aspergillus* species. The complexity of mold identification using MALDI-TOF MS systems arises in part from morphological variability, such as the presence of both hyphae and conidia, which can lead to inconsistent spectral profiles and complicate species-level identification (38). Notably, current protein extraction protocols involving chemical lysis, bead beating, and ultrasonication (39) have improved protein yield; however, challenges remain in achieving consistent species-level identification.

Although MALDI-TOF MS demonstrated promising performance in identifying several *Aspergillus* species, our study showed that PCR-sequencing of different genetic loci—ITS, β-tub, and CaM—offered higher taxonomic resolution. ITS and β-tub exhibited greater consistency and reliability, with species-level concordance rates of 88.3% and 81.1%, respectively, while CaM showed lower agreement (68.4%) and limited discriminatory power for certain species, reducing its utility as a standalone marker. Notably, in the *Tanneri* section where MALDI-TOF MS failed to yield reliable identifications, β-tub sequencing successfully resolved all isolates, underscoring the importance of molecular methods in detecting rare or cryptic taxa. Comparative analysis across these markers revealed substantial genetic variability among isolates, reinforcing the value of a multilocus approach—particularly in tertiary care settings where precise identification is essential for guiding targeted antifungal therapy (40). This approach aligns with previous studies that advocate for the use of multilocus sequencing, primarily ITS, β-tub, and CaM, to differentiate closely related species within the genus *Aspergillus* to overcome the limitation of relying on a single genetic marker for accurate identification (41, 42).

The antifungal susceptibility testing highlighted broad-spectrum antifungal activity across most agents but revealed significant intra- and inter-species MIC variability—an important consideration for clinical treatment strategies. Olorofim demonstrated outstanding *in vitro* activity against all *Aspergillus* isolates, including cryptic and multidrug-resistant strains, with geometric mean MICs ranging from 0.016 to 0.25 µg/mL, indicating its promise as a therapeutic agent. Mcafungin also showed robust activity, supporting its role in combination therapy with other antifungals. Among the triazoles, POS demonstrated the most consistent activity. However, reduced susceptibility was noted in isolates from sections *Clavati* and *Usti*, particularly *A. calidoustus*, which exhibited high geometric mean MICs across azoles and AmB. These results are consistent with previous studies reporting intrinsic azole resistance in *A. calidoustus* and related species (43). Cryptic species such as *A. lentulus*, *A. felis*, and *A. pseudoviridinutans* also showed elevated MICs to ITC, VRC, and AmB (GM MICs ranging from 3.3 to 9.8 µg/mL), consistent with multiple reports highlighting the clinical relevance of these species due to their reduced susceptibility to the tested antifungal agents (44, 45). This reduced susceptibility poses challenges for empirical azole therapy, especially when species-level identification is unavailable, increasing the risk of treatment failure. Furthermore, azole resistance was also observed in some *A. fumigatus sensu stricto* isolates, potentially reflecting prior antifungal exposure or environmental resistance selection (46, 47), but this finding requires further investigation. Amphotericin B exhibited variable activity, with some isolates from sections *Usti* (*A. puniceus*, *A. calidoustus*) and *Fumigati* (*A. lentulus*) demonstrating high MICs, consistent with earlier susceptibility studies (44, 48). In this study, TRB demonstrated variable *in vitro* activity against *Aspergillus* species, particularly those within sections *Fumigati* and *Usti*. Studies have shown that *A. calidoustus* exhibits reduced susceptibility to TRB, with MICs ranging from 0.25 to 1 µg/mL. Similarly, *A. lentulus*, a cryptic species within section *Fumigati*, has been associated with decreased susceptibility to multiple antifungal agents, including TRB (49). These findings underscore the limited utility of TRB as monotherapy in the treatment of diseases caused by these species.

The observed diversity and resistance profile of *Aspergillus* species underscores the critical importance of routine species-level identification and susceptibility testing—particularly in high-risk patient populations. The detection of highly resistant strains such as *A. tanneri* and *A. calidoustus*, both associated with poor outcomes, further supports the need for diagnosis and aggressive antifungal management—potentially involving combination regimens or newer agents—may be necessary in select cases. Moreover, in the absence of established clinical breakpoints for many cryptic species, extrapolation from *A. fumigatus* breakpoints may underestimate resistance, highlighting the urgent need for updated epidemiologic cutoff values and clinical guidelines tailored to emerging species. Finally, our data support the inclusion of new-generation antifungals, such as OLF, in future treatment frameworks, particularly for cases involving resistant or difficult-to-treat *Aspergillus* infections.

It is important to acknowledge that our study is based on data from the NIH, a highly specialized tertiary‐care institution, and its patient population is not representative of most hospitals. As a referral center, the NIH frequently receives cases that are more complex or refractory to prior therapy than might be encountered in community or general hospital settings. This referral bias likely influences our observed species distribution. Institutions such as the NIH often manage patients with advanced underlying disease, more prior interventions, and a higher proportion of “difficult-to-treat” infections—factors that may not be representative of the broader patient population. Moreover, because mortality is expressed as crude mortality in our analysis, it may reflect the severity of underlying disease and referral complexity rather than a direct association with a particular *Aspergillus* species. As such, generalization of species distributions, antifungal susceptibility patterns, or outcome associations from our cohort to non-tertiary settings should be made with caution.

### Conclusion

Our findings underscore the growing clinical significance of diverse and often cryptic *Aspergillus* species, many of which exhibit reduced susceptibility to conventional antifungal therapies.

Our findings highlight the increasing clinical importance of diverse and often cryptic *Aspergillus* species, many of which show reduced susceptibility to conventional antifungal agents. While antifungal susceptibility testing remains the primary tool for guiding therapy, accurate species-level identification provides valuable predictive information regarding likely resistance patterns and intrinsic drug tolerance. Furthermore, the development of comprehensive, species-specific treatment algorithms is crucial for optimizing therapeutic strategies, a goal that will require additional clinical outcome studies. Emerging antifungal agents such as OLF and fosmanogepix—currently in clinical development—hold promise due to their broad-spectrum activity; however, their real-world efficacy requires further validation through clinical trials. Ongoing surveillance and advancement in diagnostic strategies are also critical to optimizing outcomes in immunocompromised patients affected by aspergillosis. Moreover, this study underscores the complex and often challenging nature of *Aspergillus* infections in patients with diverse underlying diseases, highlighting the need for comprehensive mycological assessment. The development of species-specific treatment algorithms is crucial, and for high-risk populations, such as those with CGD, strategies that include correction or support of the underlying immune deficit are important.

## SUPPLEMENTAL MATERIAL

Supplemental figures aac.01184-25-s0001.docx Fig. S1 to S3.

Supplemental tables aac.01184-25-s0002.docx Tables S1 to S5.

ASM does not own the copyrights to Supplemental Material that may be linked to, or accessed through, an article. The authors have granted ASM a non-exclusive, world-wide license to publish the Supplemental Material files. Please contact the corresponding author directly for reuse.
