# Supplementary material for: Beyond Fumigatus: a molecular portrait of clinical Aspergillus diversity, pathogenicity, and antifungal resistance
Source: Antimicrob Agents Chemother. 2026 Jan 13;70(2):e01184-25. doi: 10.1128/aac.01184-25 (PMC12888877; doi:10.1128/aac.01184-25)
Supplement: Supplemental figures — Fig. S1 to S3. [file aac.01184-25-s0001.docx]

**Supplemenray** **Figure 1.** Distribution of Clinical *Aspergillus* Isolates by Specimen Type.


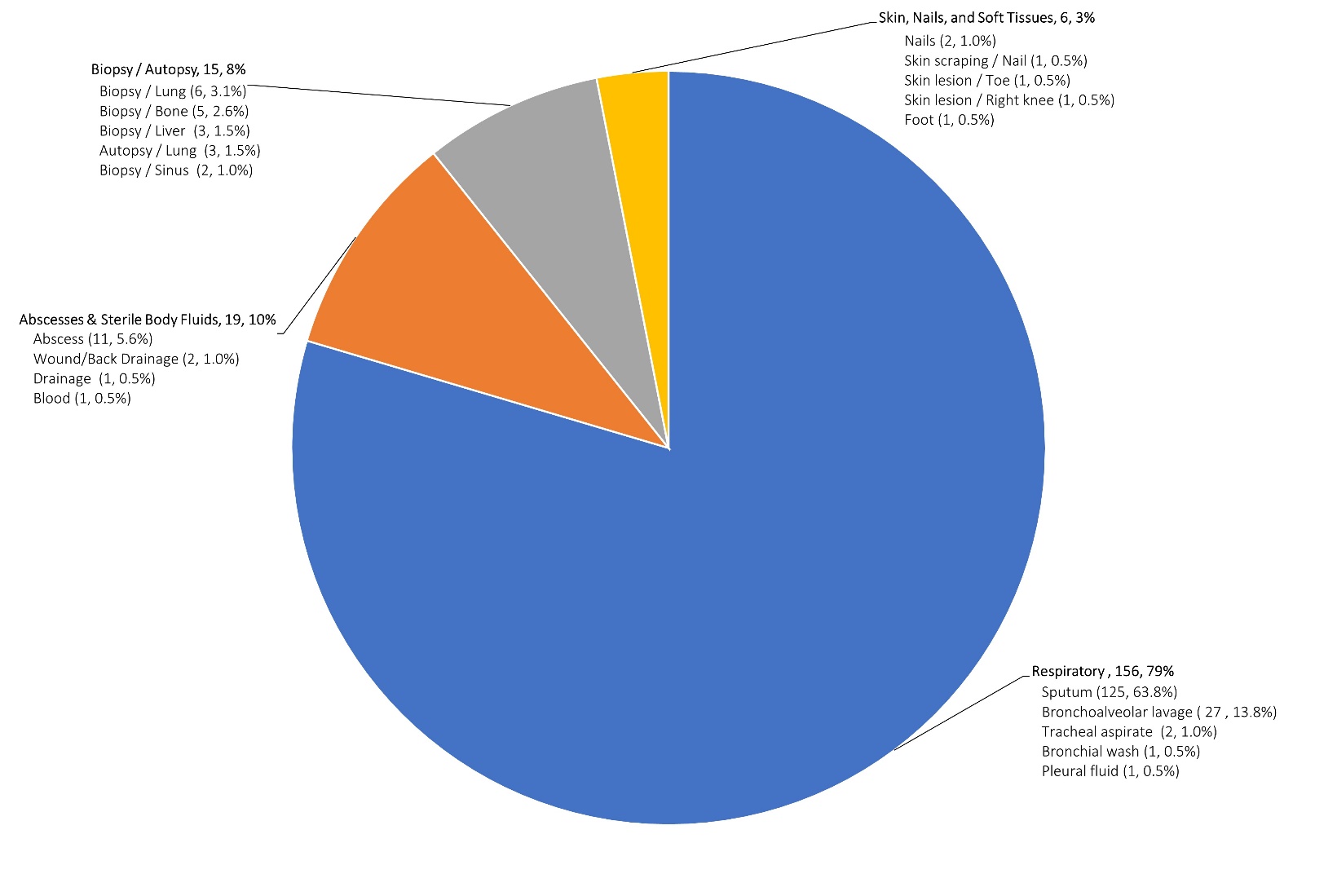


**Supplemenray Figure 2.** Source Distribution of 196 Clinical *Aspergillus* Isolates by Patient’s Underlying Condition.


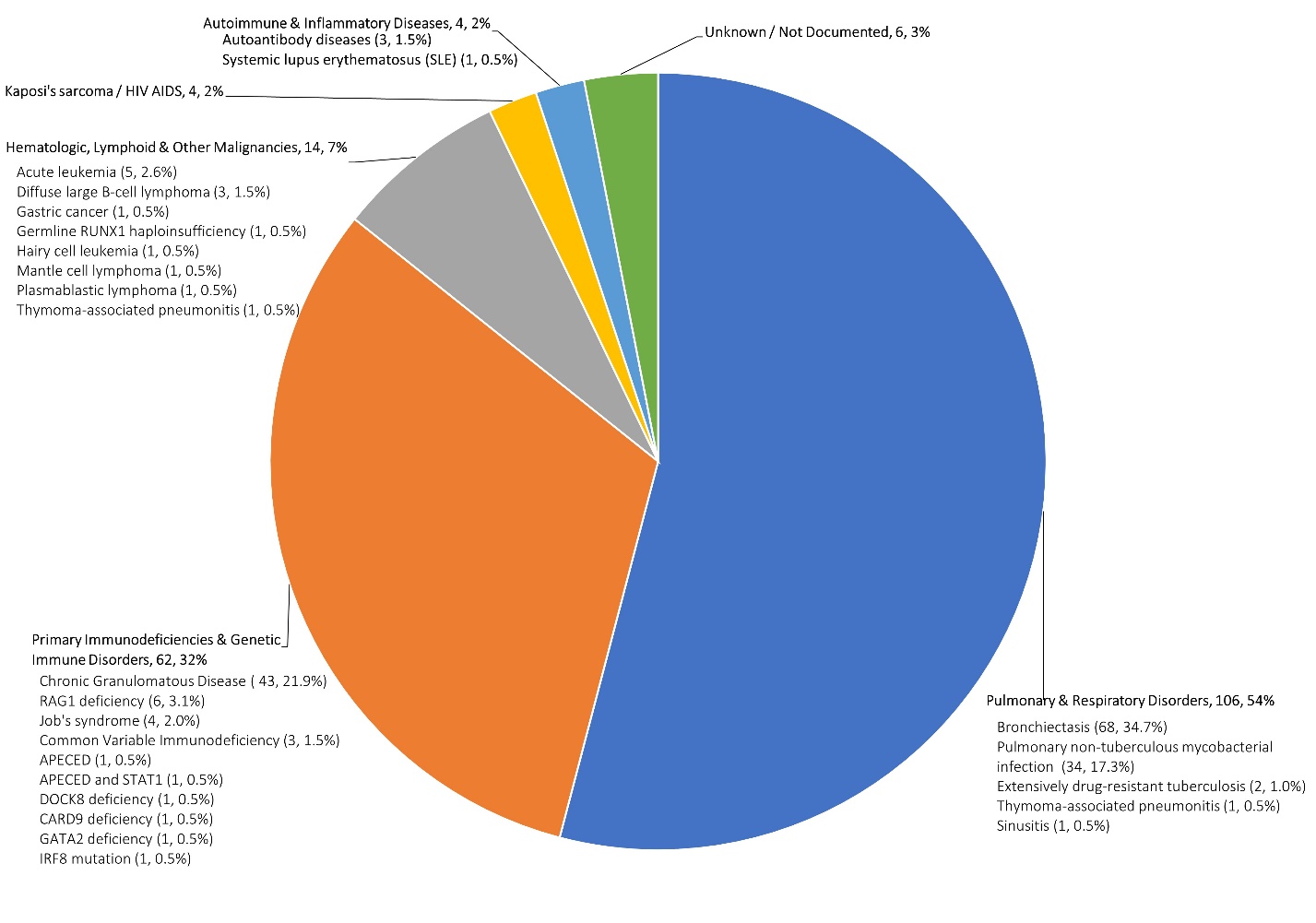


**Supplemenray** **Figure 3.** Distribution of isolates within Sections Fumigati (5A), *Nidulantes*(5B), Usti(5C), Nigri(5D).


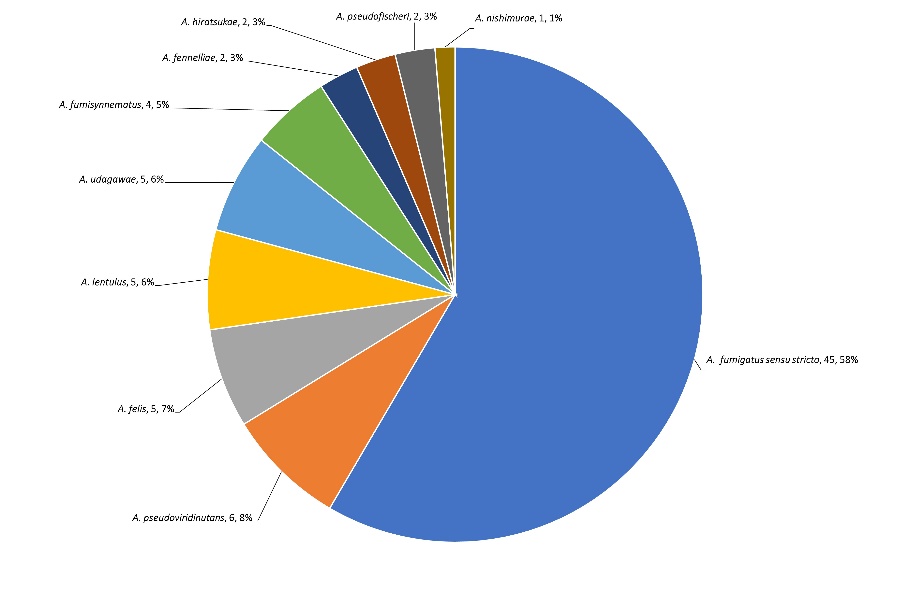

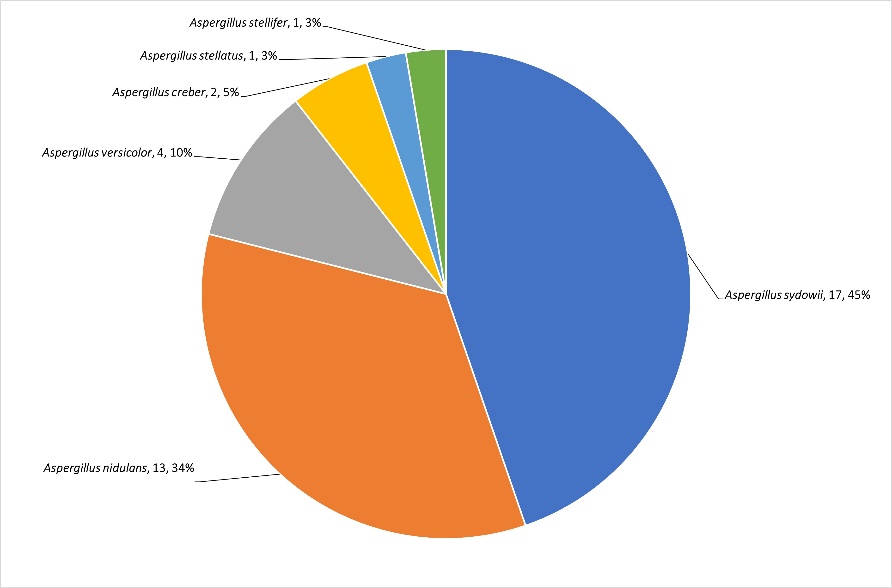

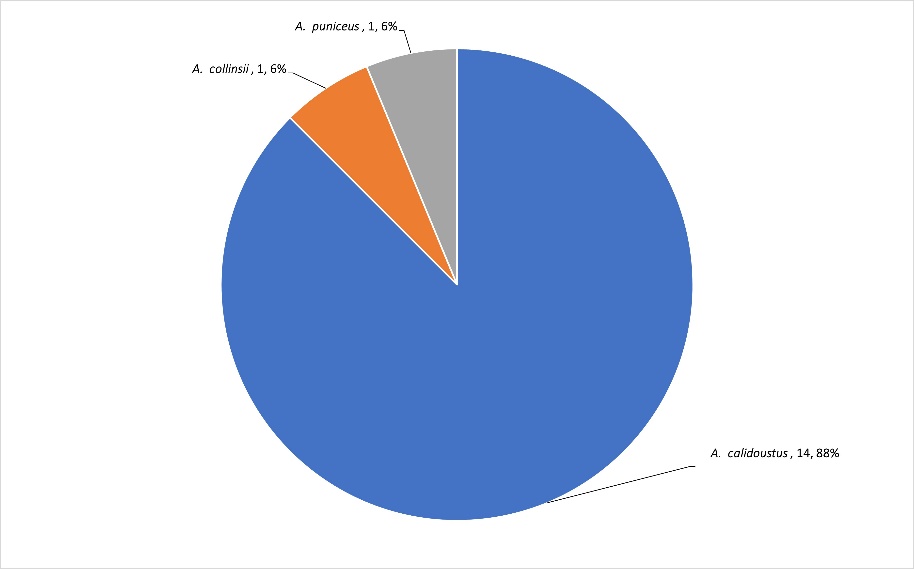

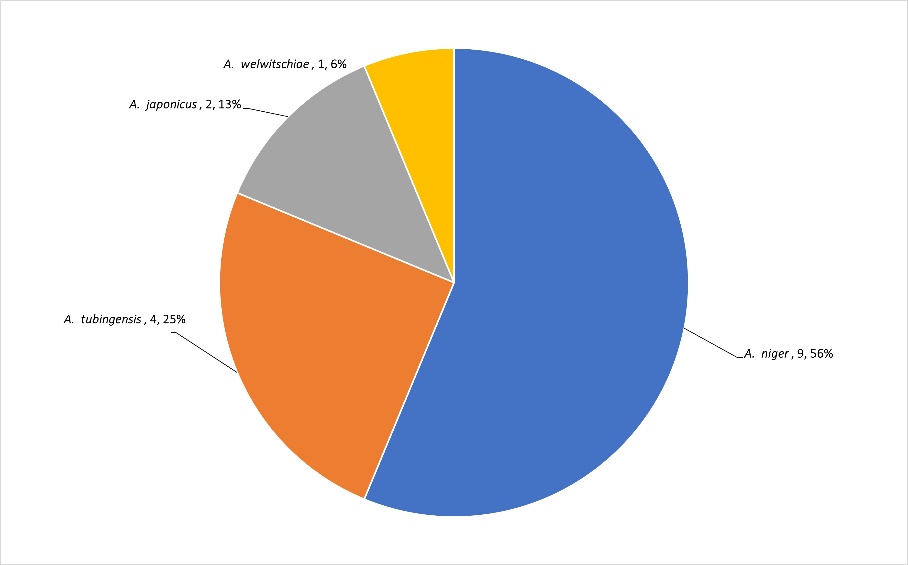


A.

C.

B.

D.
