# Supplementary material for: Beyond Fumigatus: a molecular portrait of clinical Aspergillus diversity, pathogenicity, and antifungal resistance
Source: Antimicrob Agents Chemother. 2026 Jan 13;70(2):e01184-25. doi: 10.1128/aac.01184-25 (PMC12888877; doi:10.1128/aac.01184-25)
Supplement: Supplemental tables — Tables S1 to S5. [file aac.01184-25-s0002.docx]

**Supplementary Table 1.** Clinical and molecular characteristics of *Aspergillus* isolates, including patient conditions, specimen sources, species identification, and GenBank accession numbers. APCED: Autoimmune Polyendocrinopathy-Candidiasis-Ectodermal Dystrophy; BAL: Bronchoalveolar lavage; CGD: Chronic Granulomatous Disease; DLBCL: Diffuse Large B-cell Lymphoma; CVID: Common Variable Immunodeficiency; Pulmonary NTM: Pulmonary non-tuberculous mycobacterial infection; XDR-TB: Extensively drug-resistant tuberculosis.

| **Section** | **Isolate #** | **Gene bank Accession number** | | | **Source/Site** | **Underlying medical condition** | **Outcome** | **Identification** |
| --- | --- | --- | --- | --- | --- | --- | --- | --- |
|  |  | **ITS** | **BenA** | **CmD** |  |  |  |  |
| ***Aspergillus*** | SM- 748 | PP717822 | PP812394 | PV951857 | Sputum | GATA2 deficiency | Discharged | *A. pseudoglaucaus* |
|  | SM-1459 | PX139272 | PX056268 | PX102560 | Sputum | Acute Myeloid Leukemia | Discharged | *A. pseudoglaucus* |
|  | SM-1521 | PP717823 | PV916885 | PV951804 | Sputum | Autoantibody diseases | Discharged | *A. montevidensis* |
| ***Circumdati*** | SM-915 | PP701903 | PP812395 | PX056263 | Sputum | Pulmonary NTM | Discharged | *A. ochraceus* |
|  | SM-1490 | PP701902 | PP812396 | PV951801 | Sputum | Bronchiectasis | Discharged | *A. ochraceus* |
|  | SM-575 | PP701901 | PP812397 | PP817205 | Sputum | ND | Discharged | *A. subramanianii* |
|  | SM-1231 | PP701900 | PP812398 | PP817204 | Sputum | ND | Discharged | *A. subramanianii* |
| ***Clavati*** | SM- 760 | PP706656 | PP820829 | PV951811 | Sputum | Bronchiectasis | Discharged | *A. clavatus* |
|  | SM- 765 | PP706657 | PP820829 | PX056261 | Sputum | Pulmonary NTM | Discharged | *A. clavatus* |
|  | SM-859 | PP706658 | PP820828 | PV951812 | Sputum | Pulmonary NTM | Discharged | *A. clavatus* |
|  | SM-926 | PP706659 | PV871746 | PV951813 | Sputum | Bronchiectasis | Discharged | *A. clavatus* |
| ***Flavi*** | SM -731 | PP717829 | PV871746 | PV935519 | BAL | CGD | Discharged | *A. flavus* |
|  | SM-2273 | PP717836 | PX056267 | PV935515 | Biopsy Sinus tissue | Sinusitis | Discharged | *A. flavus* |
|  | SM-907 | PP717830 | PQ435208 | PV935518 | Sputum | Bronchiectasis | Discharged | *A. flavus* |
|  | SM-1498 | PP717831 | PQ435209 | PV935522 | Biopsy skin/right knee | Pulmonary NTM | Discharged | *A. flavus* |
|  | SM-1519 | PP717833 | PQ429043 | PV935516 | Sputum | Autoantibody diseases | Discharged | *A. flavus* |
|  | SM-1502 | PP717832 | PQ435210 | PV935521 | Sputum | Bronchiectasis | Discharged | *A. flavus* |
|  | SM-2269 | PP717835 | PQ429042 | PV935517 | Tracheal aspirate | RAG1 deficiency | Discharged | *A. flavus* |
|  | SM-2108 | PP717834 | PQ435211 | PV935520 | Sputum | Bronchiectasis | Discharged | *A. flavus* |
|  | SM-875 | PP717837 | PV877092 | PV951820 | Sputum | CGD | Discharged | *A. nomius* |
|  | SM-920 | PP717838 | PV877093 | PV951827 | Sputum | Bronchiectasis | Discharged | *A. parasiticus* |
| ***Flavipides*** | SM -761 | PP706627 | PV910665 | PV951859 | Sputum | Job's syndrome | Discharged | *A. alboluteus* |
|  | SM-927 | PP706626 | PX056266 | PX056265 | Sputum | Bronchiectasis | Discharged | *A. flavipes* |
|  | SM-930 | PP706629 | PV871761 | PV951830 | Sputum | Bronchiectasis | Discharged | *A. iizukae* |
|  | SM-908 | PP706628 | PV910671 | PV951823 | Sputum | Pulmonary NTM | Discharged | *A. spelaeus* |
| ***Fumigati*** | SM -729 | PP721331 | PX114625 | PV951847 | Pleural fluid | Acute Myeloid Leukemia | Discharged | *A. nishimurae* |
|  | SM -730 | PP725513 | PV856467 | PV935491 | Abscess/paraspinal | CGD | Died | *A. udagawae* |
|  | SM -732 | PP725507 | PV871767 | PV951848 | BAL | CGD | Discharged | *A. pseudofischeri* |
|  | SM -733 | PP725506 | PV871766 | PV951849 | BAL | CGD | Discharged | *A. pseudofischeri* |
|  | SM -735 | PP725502 | PV910658 | PV951851 | BAL | CGD | Died | *A. pseudoviridinutans* |
|  | SM -742 | PP725497 | PV877089 | PV951854 | BAL | Diffuse large B cell lymphoma | Discharged | *A. lentulus* |
|  | SM- 743 | PP725498 | PV877090 | PV951855 | BAL | Diffuse large B cell lymphoma | Discharged | *A. lentulus* |
|  | SM -745 | PP725493 | PV910662 | PV951856 | Sputum | Bronchiectasis | Discharged | *A. hiratsukae* |
|  | SM -749 | PP725514 | PV856481 | PV935492 | Sputum | Bronchiectasis | Discharged | *A. fumigatus sensu stricto* |
|  | SM-2169 | PP725492 | PV916887 | PV951805 | Biopsy liver | CGD | Died | *A. felis* |
|  | SM- 752 | PP725491 | PV871757 | PV951809 | Biopsy liver | CGD | Died | *A. felis* |
|  | SM- 415 | PP725512 | PV856482 | PV935490 | BAL | Hairy cell leukemia | Discharged | *A. fumigatus sensu stricto* |
|  | SM -754 | PP725485 | PX114626 | PV951821 | Abscess/paraspinal | CGD | Died | *A. udagawae* |
|  | SM-804 | PP725515 | PV856480 | PV935493 | Sputum | Pulmonary NTM | Discharged | *A. fumigatus sensu stricto* |
|  | SM-807 | PP725547 | PV856478 | PV935495 | Sputum | Bronchiectasis | Discharged | *A. fumigatus sensu stricto* |
|  | SM-923 | PP725494 | PV877094 | PV951828 | Sputum | Bronchiectasis | Discharged | *A. hiratsukae* |
|  | SM-862 | PP725516 | PV856466 | PV935496 | Sputum | Bronchiectasis | Discharged | *A. fumigatus sensu stricto* |
|  | SM-805 | PP725549 | PV856479 | PV935494 | Sputum | Bronchiectasis | Discharged | *A. fumigatus sensu stricto* |
|  | SM-871 | PP725517 | PV856465 | PV935497 | Sputum | Bronchiectasis | Discharged | *A. fumigatus sensu stricto* |
|  | SM-864 | PP725503 | PV910666 | PV951815 | Sputum | CGD | Died | *A. pseudoviridinutans* |
|  | SM-865 | PP725504 | PV910667 | PV951816 | Abscess/upper lobe of the lung | CGD | Died | *A. pseudoviridinutans* |
|  | SM-867 | PP725487 | PV910668 | PV951817 | Autopsy lung | CGD | Died | *A. udagawae* |
|  | SM-868 | PP725486 | PV910669 | PV951818 | Autopsy lung | CGD | Died | *A. udagawae* |
|  | SM-870 | PP725505 | PV910670 | PV951819 | Sputum | CGD | Died | *A. pseudoviridinutans* |
|  | SM- 43 | PP725484 | PV891348 | PV951839 | Sputum | Bronchiectasis | Discharged | *A. udagawae* |
|  | SM- 65 | PP725499 | PV910654 | PV951840 | Sputum | Bronchiectasis | Discharged | *A. lentulus* |
|  | SM -9 | PP725495 | PV891345 | PV951835 | Sputum | Bronchiectasis | Discharged | *A. lentulus* |
|  | SM -32 | PP725548 | PV856468 | PX096751 | Sputum | Bronchiectasis | Discharged | *A. fumigatus sensu stricto* |
|  | SM- 33 | PP725482 | PV891347 | PV951838 | Sputum | Bronchiectasis | Discharged | *A. fumisynnematus* |
|  | SM -137 | PP725496 | PX118965 | PV951844 | Sputum | Bronchiectasis | Discharged | *A. lentulus* |
|  | SM -110 | PP725501 | PV910656 | PV951843 | Sputum | CGD | Died | *A. pseudoviridinutans* |
|  | SM -138 | PP721334 | PX114627 | PV951845 | Sputum | Bronchiectasis | Discharged | *A. fumisynnematus* |
|  | SM- 112 | PP725489 | PX229959 | PV951808 | Sputum | CGD | Died | *A. felis* |
|  | SM -103 | PP725500 | PX114628 | PV951842 | Sputum | CGD | Died | *A. pseudoviridinutans* |
|  | SM- 16 | PP721333 | PX229958 | PV951836 | Sputum | Bronchiectasis | Discharged | *A. fenelliae* |
|  | SM -93 | PP725488 | PV920633 | PV951806 | Sputum | CGD | Died | *A. felis* |
|  | SM- 17 | PP721332 | PV891346 | PV951837 | Sputum | Bronchiectasis | Discharged | *A. fenelliae* |
|  | SM- 106 | PP725490 | PX229960 | PV951807 | Sputum | CGD | Died | *A. felis* |
|  | SM- 83 | PP725481 | PV910655 | PV951841 | Sputum | Bronchiectasis | Discharged | *A. fumisynnematus* |
|  | SM -142 | PP725483 | PV877088 | PV951846 | Sputum | Bronchiectasis | Discharged | *A. fumisynnematus* |
|  | SM -1369 | PP725518 | PV920632 | PX096752 | Sputum | Bronchiectasis | Discharged | *A. fumigatus sensu stricto* |
|  | SM-1460 | PP725546 | PV845737 | PX096753 | Sputum | Pulmonary NTM | Discharged | *A. fumigatus sensu stricto* |
|  | SM-1375 | PP725551 | PX229967 | PX096754 | Sputum | Bronchiectasis | Discharged | *A. fumigatus sensu stricto* |
|  | SM-1464 | PP725544 | PV845738 | PX096755 | Sputum | Bronchiectasis | Discharged | *A. fumigatus sensu stricto* |
|  | SM-1465 | PP725545 | PV845739 | PX096756 | BAL | Pulmonary NTM | Discharged | *A. fumigatus sensu stricto* |
|  | SM-1467 | PP725543 | PV845740 | PX096757 | Sputum | Bronchiectasis | Discharged | *A. fumigatus sensu stricto* |
|  | SM-1475 | PP725523 | PV845741 | PX096758 | Sputum | RAG1 deficiency | Died | *A. fumigatus sensu stricto* |
|  | SM-1476 | PP725524 | PV845742 | PX114623 | Sputum | RAG1 deficiency | Died | *A. fumigatus sensu stricto* |
|  | SM-1477 | PP725552 | PV845743 | PX118966 | Sputum | Bronchiectasis | Discharged | *A. fumigatus sensu stricto* |
|  | SM-1478 | PP725511 | PV845744 | PX096759 | Sputum | Pulmonary NTM | Discharged | *A. fumigatus* sensu stricto |
|  | SM-1479 | PP725542 | PV845745 | PX096760 | Sputum | Bronchiectasis | Discharged | *A. fumigatus sensu stricto* |
|  | SM-1483 | PP725528 | PV845746 | PX096761 | Sputum | Bronchiectasis | Discharged | *A. fumigatus sensu stricto* |
|  | SM-1484 | PP725525 | PV845747 | PX114624 | Sputum | Pulmonary NTM | Discharged | *A. fumigatus sensu stricto* |
|  | SM-1489 | PP725540 | PV845749 | PV920636 | Sputum | Pulmonary NTM | Discharged | *A. fumigatus sensu stricto* |
|  | SM-1486 | PP725541 | PV845748 | PV920634 | Sputum | Pulmonary NTM | Discharged | *A. fumigatus sensu stricto* |
|  | SM-1491 | PP725534 | PV845750 | PV920637 | Sputum | Bronchiectasis | Discharged | *A. fumigatus sensu stricto* |
|  | SM-1492 | PP725539 | PV856473 | PV920638 | Sputum | Bronchiectasis | Discharged | *A. fumigatus sensu stricto* |
|  | SM-1493 | PP725526 | PV856474 | PV920639 | Sputum | Bronchiectasis | Discharged | *A. fumigatus sensu stricto* |
|  | SM-1495 | PP725535 | PV856475 | PV920640 | Sputum | Bronchiectasis | Discharged | *A. fumigatus sensu stricto* |
|  | SM-1496 | PP725536 | PV856476 | PV920641 | Sputum | Autoantibody diseases | Discharged | *A. fumigatus sensu stricto* |
|  | SM-1497 | PP725527 | PV856477 | PV920642 | Sputum | Bronchiectasis | Discharged | *A. fumigatus sensu stricto* |
|  | SM-1501 | PP725537 | PV816235 | PV920643 | Sputum | Job's syndrome | Discharged | *A. fumigatus sensu stricto* |
|  | SM-1503 | PP728629 | PV816234 | PV920644 | Sputum | Job's syndrome | Discharged | *A. fumigatus sensu stricto* |
|  | SM-1504 | PP728630 | PV816233 | PV920645 | Sputum | Pulmonary NTM | Discharged | *A. fumigatus sensu stricto* |
|  | SM-1514 | PP725508 | PV816232 | PV920646 | Sputum | Mantle cell lymphoma | Discharged | *A. fumigatus sensu stricto* |
|  | SM-1515 | PP725509 | PV816231 | PV920647 | BAL | CARD9 deficiency | Discharged | *A. fumigatus sensu stricto* |
|  | SM-1520 | PP725510 | PV816230 | PV920648 | Sputum | Bronchiectasis | Discharged | *A. fumigatus sensu stricto* |
|  | SM-1528 | PP725519 | PV816229 | PV920649 | Sputum | Bronchiectasis | Discharged | *A. fumigatus sensu stricto* |
|  | SM-1529 | PP725520 | PV816228 | PV920635 | Sputum | Bronchiectasis | Discharged | *A. fumigatus sensu stricto* |
|  | SM-1530 | PP725521 | PV816227 | PV935484 | Sputum | Pulmonary NTM | Discharged | *A. fumigatus sensu stricto* |
|  | SM-1539 | PP725522 | PV816226 | PV935485 | Sputum | Pulmonary NTM | Discharged | *A. fumigatus sensu stricto* |
|  | SM-2109 | PP725529 | PV816225 | PV935486 | Sputum | Pulmonary NTM | Discharged | *A. fumigatus sensu stricto* |
|  | SM-2110 | PP725538 | PV816224 | PV935487 | Sputum | Bronchiectasis | Discharged | *A. fumigatus sensu stricto* |
|  | SM-2111 | PP725530 | PV816223 | PX207090 | Sputum | Bronchiectasis | Discharged | *A. fumigatus sensu stricto* |
|  | SM-2113 | PP725531 | PV816222 | PV935488 | Sputum | Bronchiectasis | Discharged | *A. fumigatus sensu stricto* |
|  | SM-2120 | PP725532 | PV816221 | PV935489 | Bronchial wash | Pulmonary NTM | Discharged | *A. fumigatus sensu stricto* |
|  | SM-2121 | PP725533 | PV816220 | PV951850 | Sputum | Bronchiectasis | Discharged | *A. fumigatus sensu stricto* |
| ***Nidulantes*** | SM -734 | PP701913 | PX229961 | PV951850 | BAL | Acute lymphoid leukemia | Discharged | *A. nidulans* |
|  | SM -740 | PP717071 | PV871765 | PV951853 | Sputum | Bronchiectasis | Discharged | *A. versicolor* |
|  | SM -741 | PP702198 | PV752207 | PV935501 | BAL | Pulmonary NTM | Discharged | *A. sydowii* |
|  | SM- 744 | PP702204 | PV752208 | PV935502 | Sputum | Bronchiectasis | Discharged | *A. sydowii* |
|  | SM- 751 | PP706314 | PV871756 | PV951810 | Biopsy /liver | CGD | Died | *A. creber* |
|  | SM -438 | PP702197 | PV711359 | PV935500 | Sputum | Pulmonary NTM | Discharged | *A. sydowii* |
|  | SM -762 | PP702203 | PV752209 | PV935503 | Skin lesion/Toe | Kaposi's sarcoma/HIV AIDS | Discharged | *A. sydowii* |
|  | SM -763 | PP717072 | PV871764 | PX056260 | Sputum | Job's syndrome | Discharged | *A. versicolor* |
|  | SM-766 | PP702202 | PV786151 | PV935504 | BAL | Combined immunodeficiency due to RAG1 deficiency | Discharged | *A. sydowii* |
|  | SM-857 | PP702199 | PV786152 | PV935505 | Sputum | Pulmonary NTM | Discharged | *A. sydowii* |
|  | SM-858 | PP702200 | PV786153 | PV935506 | Sputum | Bronchiectasis | Discharged | *A. sydowii* |
|  | SM-863 | PP701914 | PX229962 | PV951814 | Biopsy / Foot | CGD | Discharged | *A. nidulans* |
|  | SM-869 | PP702201 | PV786154 | PV935507 | Sputum | Pulmonary NTM | Discharged | *A. sydowii* |
|  | SM-905 | PP717073 | PV871763 | PV951822 | Sputum | Bronchiectasis | Discharged | *A. versicolor* |
|  | SM-906 | PP702205 | PV786155 | PV935510 | BAL | DLBCL | Discharged | *A. sydowii* |
|  | SM-909 | PP701915 | PX056273 | PV951824 | Biopsy lung | CGD | Discharged | *A. nidulans* |
|  | SM-910 | PP701916 | PX229964 | PV951825 | Biopsy lung | CGD | Discharged | *A. nidulans* |
|  | SM-911 | PP701917 | PX056271 | PX207091 | Biopsy lung | CGD | Discharged | *A. nidulans* |
|  | SM-912 | PX229852 | PX229965 | PX207092 | Biopsy lung | CGD | Discharged | *A. nidulans* |
|  | SM-913 | PP701918 | PX056270 | PV951826 | Biopsy lung | CGD | Discharged | *A. nidulans* |
|  | SM-918 | PP702206 | PV786156 | PV935511 | BAL | ND | Discharged | *A. sydowii* |
|  | SM-924 | PP706315 | PP820830 | PX056264 | Nails | DOCK8 deficiency | Discharged | *A. creber* |
|  | SM-925 | PP717074 | PV871762 | PV951829 | Nails | ND | Discharged | *A. versicolor* |
|  | SM-928 | PP701919 | PV871747 | PX207093 | Sputum | Bronchiectasis | Discharged | *A. nidulans* |
|  | SM -1368 | PP701920 | PX229966 | PV871745 | Biopsy rib bone | CGD | Discharged | *A. nidulans* |
|  | SM-1373 | PP702209 | PV786157 | PV935509 | BAL | Pulmonary NTM | Discharged | *A. sydowii* |
|  | SM-1463 | PP702207 | PV786158 | PV935508 | BAL | Pulmonary NTM | Discharged | *A. sydowii* |
|  | SM-1376 | PP701921 | PX229968 | PV951803 | Biopsy rib bone | CGD | Discharged | *A. nidulans* |
|  | SM-1494 | PP701923 | PV916895 | PV951802 | Sputum | Bronchiectasis | Discharged | *A. nidulans* |
|  | SM-1499 | PP702210 | PV786159 | PV935498 | Sputum | Pulmonary NTM | Discharged | *A. sydowii* |
|  | SM-1481 | PP701922 | PV916892 | PX207094 | Sputum | Bronchiectasis | Discharged | *A. nidulans* |
|  | SM-1510 | PP702208 | PV786160 | PV935499 | Sputum | Bronchiectasis | Discharged | *A. sydowii* |
|  | SM-2185 | PP702211 | PV786161 | PV935512 | Sputum | Pulmonary NTM | Discharged | *A. sydowii* |
|  | SM-2186 | PP702212 | PV786162 | PV935513 | BAL | Thymoma-associated pneumonitis | Discharged | *A. sydowii* |
|  | SM-2187 | PP701924 | PX056269 | PV951831 | Abscess/right chest | CGD | Discharged | *A. nidulans* |
|  | SM-2271 | PP702213 | PV786163 | PV935514 | BAL | Pulmonary NTM | Discharged | *A. sydowi* |
|  | SM-2275 | PP706385 | PV916889 | PV951832 | Sputum | Bronchiectasis | Discharged | *A. stellatus* |
|  | SM-2272 | PP706317 | PV916888 | PV951834 | Sputum | Bronchiectasis | Discharged | *A. stellifer* |
| **Section Nigri** | SM- 736 | PP718796 | PV910659 | PV935531 | Sputum | Kaposi's sarcoma/HIV AIDS | Discharged | *A. tubingensis* |
|  | SM -758 | PP718788 | PV871749 | PV935527 | Skin scraping/Nail | ND | Discharged | *A. niger* |
|  | SM -759 | PP718787 | PV910664 | PV935528 | Sinus/right maxilla | Common Variable Immunodeficiency | Discharged | *A. niger* |
|  | SM -764 | PP718789 | PX118962 | PX102561 | BAL | Acute Lymphoblastic Leukemia | Discharged | *A. niger* |
|  | SM-919 | PP718790 | PV871750 | PV935529 | BAL | ND | Discharged | *A. niger* |
|  | SM-931 | PP718791 | PV871751 | PV935530 | BAL | HIV AIDS | Discharged | *A. niger* |
|  | SM-1371 | PP718802 | PV871760 | PV935535 | BAL | APCED | Discharged | *A. welwitschiae* |
|  | SM-929 | PP718801 | PV916890 | PX114618 | Sputum | THYMOMA | Discharged | *A. japonicus* |
|  | SM-1466 | PP718792 | PV871752 | PV935526 | Sputum | Bronchiectasis | Discharged | *A. niger* |
|  | SM-1469 | PP718793 | PV871753 | PV935525 | Sputum | Bronchiectasis | Discharged | *A. niger* |
|  | SM-1480 | PP718794 | PV871754 | PV935524 | Sputum | Pulmonary NTM | Discharged | *A. niger* |
|  | SM-1482 | PP718800 | PV916893 | PV935536 | Sputum | Bronchiectasis | Discharged | *A. japonicus* |
|  | SM-1487 | PP718795 | PV871755 | PV935523 | Sputum | Bronchiectasis | Discharged | *A. niger* |
|  | SM-1488 | PP718798 | PV916894 | PV935534 | Sputum | Bronchiectasis | Discharged | *A.tubingensis* |
|  | SM-1500 | PP718797 | PV916884 | PV935533 | Sputum | CVID | Discharged | *A. tubingensis* |
|  | SM-1527 | PP718799 | PV916886 | PV935532 | Sputum | XDR-TB | Discharged | *A. tubingensis* |
| ***Tanneri*** | SM- 755 | PP718685 | PV871758 | ND | Autopsy lung | CGD | Died | *A. tanneri* |
|  | SM- 1327 | PP706681 | PX118964 | ND | Drainage | CGD | Died | *A. tanneri* |
|  | SM -756 | PP718682 | PV856495 | ND | Abscess/frontotemporal | CGD | Died | *A. tanneri* |
|  | SM- 757 | PP718680 | PV856496 | ND | Biopsy / left frontal lobe lesion | CGD | Died | *A. tanneri* |
|  | SM-873 | PP718681 | PV856497 | ND | ABSCESS/R perinephric abscess | CGD | Died | *A. tanneri* |
|  | SM-878 | PP718686 | PV856498 | ND | Wound/back drainage | CGD | Died | *A. tanneri* |
|  | SM- 25 | PP718684 | PV856491 | ND | Abscess | CGD | Died | *A. tanneri* |
|  | SM -26 | PP718679 | PV856492 | ND | Abscess | CGD | Died | *A. tanneri* |
|  | SM- 28 | PP718678 | PV856493 | ND | Abscess | CGD | Died | *A. tanneri* |
|  | SM- 38 | PP718683 | PV856494 | ND | Abscess | CGD | Died | *A. tanneri* |
|  | SM- 1370 | PP718687 | PV871759 | ND | Wound/back drainage | CGD | Died | *A. tanneri* |
|  | SM -1326 | PP725550 | PX118963 | ND | ABSCESS/R perinephric abscess | CGD | Died | *A. tanneri* |
| ***Terrei*** | SM -738 | PP706674 | PP848162 | PV951852 | Sputum | Pulmonary NTM | Discharged | *A. terreus* |
|  | SM-806 | PP706675 | PP848170 | PP852691 | Sputum | Bronchiectasis | Discharged | *A. terreus* |
|  | SM-861 | PP706678 | PP848163 | PP852692 | BAL | Systemic lupus erythematosus | Discharged | *A. terreus* |
|  | SM-872 | PP706677 | PX229963 | PX114621 | Sputum | Pulmonary NTM | Discharged | *A. terreus* |
|  | SM-874 | PP706679 | PP848164 | PP852693 | Sputum | Pulmonary NTM | Discharged | *A. terreus* |
|  | SM-877 | PP706676 | PP848165 | PP852694 | Blood | Gastric cancer | Discharged | *A. terreus* |
|  | SM-904 | PP706680 | PP848166 | PP852695 | Biopsy bone | CGD | Discharged | *A. terreus* |
|  | SM 1537 | PP706682 | PP848167 | PP852697 | Sputum | Bronchiectasis | Discharged | *A. terreus* |
|  | SM-1472 | PP706683 | PP848171 | PP852696 | Sputum | Pulmonary NTM | Discharged | *A. terreus* |
|  | SM-2176 | PP706684 | PP848168 | PP852698 | Sputum | Pulmonary NTM | Discharged | *A. terreus* |
|  | SM-2270 | PP706685 | PP848169 | PP852699 | Sputum | Bronchiectasis | Discharged | *A. terreus* |
|  | SM-2274 | PP706686 | PP852700 | PV951833 | Sputum | XDR-TB | Discharged | *A. niveus* |
| ***Usti*** | SM -737 | PP718734 | PQ429044 | PV951790 | Sputum | Bronchiectasis | Discharged | *A. calidoustus* |
|  | SM- 739 | PP718735 | PV910661 | PV951860 | BAL | Kaposi's sarcoma | Discharged | *A. calidoustus* |
|  | SM- 746 | PP718736 | PV910663 | PV951791 | Sputum | RAG1 deficiency | Died | *A. calidoustus* |
|  | SM- 747 | PP718737 | PV856470 | PV951792 | Tracheal Aspirate | RAG1 deficiency | Died | *A. calidoustus* |
|  | SM -750 | PP718749 | PV871748 | PV951858 | Sputum | IRF8 mutation | Discharged | *A. puniceus* |
|  | SM -753 | PP718738 | PV856490 | PV951793 | Sputum | CGD | Died | *A. calidoustus* |
|  | SM-866 | PP718739 | PV856489 | PV951794 | Sputum | Common Variable Immunodeficiency | Discharged | *A. calidoustus* |
|  | SM-876 | PP718740 | PV856488 | PV951795 | Sputum | Pulmonary NTM | Discharged | *A. calidoustus* |
|  | SM-917 | PP718741 | PV856487 | PV951800 | BAL | APCED and STAT1 | Discharged | *A. calidoustus* |
|  | SM-1372 | PP718746 | PV856486 | PV951796 | BAL | Bronchiectasis | Discharged | *A. calidoustus* |
|  | SM-1374 | PP718742 | PV856485 | PV951799 | Sputum | Bronchiectasis | Discharged | *A. calidoustus* |
|  | SM-1377 | PP718743 | PV856484 | PV951798 | Biopsy left maxilla | Plasmablastic lymphoma | Discharged | *A. calidoustus* |
|  | SM-1461 | PP718744 | PV916891 | PX056259 | Sputum | Pulmonary NTM | Discharged | *A. calidoustus* |
|  | SM-1485 | PP718745 | PV856483 | PV951797 | Sputum | Bronchiectasis | Discharged | *A. calidoustus* |
|  | SM-2163 | PP718747 | PQ429045 | PV951789 | Biopsy lung | Acute leukemia | Discharged | *A. calidoustus* |
|  | SM-860 | PP718748 | PV877091 | PX056262 | BAL | Germline RUNX1 haploinsufficiency | Discharged | *A. collinsii* |

**Supplementary Table 2.** Primer sequences used for PCR amplification and sequencing of internal transcribed spacer (ITS) region of rDNA, β-tubulin, and calmodulin genes for *Aspergillus* species identification.

| **Locus** | **Primer** | **Sequence (5^'^ - 3^'^)** | **Size (bp)** | **References** |
| --- | --- | --- | --- | --- |
| Internal Transcribed Spacer (ITS) of ribosomal RNA (rRNA) | ITS1_F | TCC GTA GGT GAA CCT GCG G | 565-613 | Samson et al. 2014 |
|  | ITS4_R | TCC TCC GCT TAT TGA TAT GC |  |  |
| Partial β-tubulin (*tubb*) | Bt2a_F | GGT AAC CAA ATC GGT GCT TTC | 432-600 |  |
|  | Bt2b_R | ACC CTC AGT GTA GTG ACC CTT GGC |  |  |
| Partial calmodulin (*CaM*) | CMD5_F | CCG AGT ACA AGG AGG CCT TC | 500-600 |  |
|  | CMD6_R | CCG ATR GAG GTC ATA ACG TGG |  |  |

**Supplementary Table 3.** Conidial inoculum densities, antifungal concentration ranges, and incubation times used for susceptibility testing, including minimum inhibitory concentration (MIC) for all antifungals except micafungin, for which minimum effective concentration (MEC) values were determined.

|  | Densities of the conidial suspensions inoculated (adjusted to an absorbance at 530 nm) | Range of Concentrations Tested (µg/mL) | Incubation times for determining MIC /MEC (hours) |
| --- | --- | --- | --- |
| AmB - amphotericin B | 0.9 to 0.13 | 0.016 to 16 | 48 |
| ITC – itraconazole |  |  | 48 |
| VRC – voriconazole |  |  | 48 |
| POSA – posaconazole |  |  | 48 |
| ISA – isavuconazole |  |  | 48 |
| TRB – terbinafine |  |  | 48 |
| MFG – micafungin |  |  | 24 |
| OLF – olorofim |  |  | 48 |

**Supplementary Table 4.** Distribution of the Identified Aspergillus Species by Specimen Type

| **Respiratory (n=156)** | | |
| --- | --- | --- |
| Sputum | 125 | *A. fumigatus sensu stricto* (41), *A. calidoustus (8), A. sydowii* (8)*, A. terreus (8), A. pseudoviridinutans* (4), *A. fumisynnematus (4), A. clavatus (4), A. flavus (4), A. tubingensis (4), A. felis (3), A. lentulus (3), A. nidulans (3), A. niger (4), A. versicolor (3), A. fenelliae (2), A. hiratsukae (2), A. japonicus (2), A. pseudoglaucus* (2)*, A. ochraceus (2)*, *A. subramanianii (2), A montevidensis* (1), *A. parasiticus (1), A. nomius* (1), *A. niveus* (1)*, A. stellatus* (1), *A. stellifer (1), A. puniceus* (1), *A. udagawae* (1), *A. flavipes* (1), *A. iizukae* (1), *A. alboluteus* (1), *A. spelaeus* (1) |
| Bronchoalveolar lavage (BAL) | 27 | *A. sydowii (8), A. calidoustus (3), A niger (3), A. pseudofischeri* (2), *A. lentulus* (2), *A. pseudoviridinutans* (1), A. flavus (1), A. nidulans (1), *A. welwitschiae* (1), *A. collinsii* (1), *A. terreus* (1) |
| Tracheal aspirate | 2 | *A. flavus* (1), *A. calidoustus* (1) |
| Bronchial wash | 1 | *A. fumigatus sensu stricto* |
| Pleural fluid | 1 | *A. nishimurae* |
| **Biopsy / Autopsy (n=19)** | | |
| Biopsy lung | 6 | A. nidulans (5), *A. calidoustus* (1) |
| Biopsy bone | 5 | *A. nidulans* (2), *A. calidoustus* (1),  *A. terreus* (1), *A. tanneri* (1) |
| Biopsy liver | 3 | *A. felis* (2), *A. creber* (1) |
| Autopsy lung | 3 | *A. udagawae* (2), *A. tanneri* (1) |
| Biopsy Sinus | 2 | *A. flavus* (1), *A. niger* (1) |
| **Abscesses & Sterile Body Fluids (n=15)** | | |
| Abscess | 11 | *A. tanneri* (7), *A. udagawae* (2), *A. pseudoviridinutans* (1), *A. nidulans* (1) |
| Drainage | 1 | *A. tanneri* |
| Wound/back drainage | 2 | *A. tanneri* (2) |
| Blood | 1 | *A. terreus* |
| **Skin, Nails, and Soft Tissue (n=6)** | | |
| Nails | 2 | *A. creber* (1), *A. versicolor* (1) |
| Skin scraping/Nail | 1 | *A. niger* |
| Skin lesion/Toe | 1 | A. sydowii |
| Skin lesion/right knee | 1 | A. flavus |
| Foot | 1 | *A. nidulans* |

**Supplementary Table 5.** Distribution of the Identified Aspergillus Species by Underlying Patient Condition.

| **Pulmonary & Respiratory Disorders (n=106)** | | |
| --- | --- | --- |
| **Bronchiectasis** | 68 | *A. fumigatus sensu stricto* (25), *A. calidoustus* (4), *A. fumisynnematus* (4), *A. flavus* (3), *A. lentulus* (3), *A. nidulans* (3), *A. niger* (3), *A. sydowii* (3), *A. terreus* (3), *A. clavatus* (2), *A. fenelliae* (2), *A. hiratsukae* (2), *A. versicolor* (2), A. flavipes (1), *A. iizukae* (1), *A. japonicus* (1), *A. ochraceus* (1), *A. parasiticus* (1), *A. stellatus* (1), *A. stellifer* (1), *A. tubingensis* (1), *A. udagawae* (1) |
| **Pulmonary non-tuberculous mycobacterial infection (Pulmonary NTM)** | 34 | *A. fumigatus sensu stricto* (12), *A. sydowii* (9), *A. terreus* (5), *A. calidoustus* (2), *A. clavatus* (2), *A. niger* (1), *A. flavus* (1), *A. spelaeus* (1), *A. ochraceus* (1) |
| **Extensively drug-resistant tuberculosis (XDR-TB)** | 2 | *A. niveus* (1), *A. tubingensis* (1) |
| **Thymoma-associated pneumonitis** | 1 | *Aspergillus sydowii* |
| **Sinusitis** | 1 | *Aspergillus flavus* |
| **Primary Immunodeficiencies & Genetic Immune Disorders (n=62)** | | |
| **Chronic Granulomatous Disease (CGD)** | 43 | *A. tanneri* (12), *A. nidulans* (9), *A. pseudoviridinutans* (6), *A. felis* (5), *A. udagawae* (4), *N. pseudofischeri* (2), *A. calidoustus* (1), *A. creber* (1), *A. flavus* (1), *A. nomius* (1), *A. terreus* (1) |
| **RAG1 deficiency** | 6 | *A. calidoustus* (2), *A. fumigatus sensu stricto* (2), *A. flavus* (1), *A. sydowii* (1) |
| **Job's syndrome** | 4 | *A. fumigatus sensu stricto* (2), *A. alboluteus* (1), *A. versicolor* (1) |
| **Common Variable Immunodeficiency (CVID)** | 3 | *A. calidoustus* (2), *A. sydowii* (1) |
| **Autoimmune Polyendocrinopathy-Candidiasis-Ectodermal Dystrophy (APECED)** | 1 | *Aspergillus welwitschiae* |
| **APECED and STAT1 Gain-of-Function mutations** | 1 | *Aspergillus calidoustus* |
| **DOCK8 deficiency** | 1 | *Aspergillus creber* |
| **CARD9 deficiency** | 1 | *Aspergillus fumigatus sensu stricto* |
| **GATA2 deficiency** | 1 | *Aspergillus pseudoglaucaus* |
| **IRF8 mutation** | 1 | *Aspergillus puniceus* |
| **Hematologic, Lymphoid & Other Malignancies (n=14)** | | |
| **Diffuse Large B-cell Lymphoma (DLBCL)** | 3 | *A. lentulus* (2), *A. sydowii (*1) |
| **Acute leukemia** | 5 | *A. calidoustus* (1), *A. niger* (1), *A. nidulans* (1), *A. nishimurae* (1), *A. pseudoglaucus* (1) |
| **Gastric cancer** | 1 | *Aspergillus terreus* |
| **Germline RUNX1 haploinsufficiency** | 1 | *Aspergillus collinsii* |
| **Hairy cell leukemia** | 1 | *Aspergillus fumigatus* sensu stricto |
| **Mantle cell lymphoma** | 1 | *Aspergillus fumigatus sensu stricto* |
| **Plasmablastic lymphoma** | 1 | *Aspergillus calidoustus* |
| **Thymoma-associated pneumonitis** | 1 | *Aspergillus japonicus* |
| **Unknown / Not Documented (n=6)** | | |
| **ND (Not Documented)** | 6 | *A. niger* (2), *A. subramanianii* (2), *A. sydowii* (1), *A. versicolor* (1) |
| **Autoimmune & Inflammatory Diseases (n=4)** | | |
| **Autoantibody diseases** | 3 | *A. flavus* (1), *A. fumigatus sensu stricto* (1), *A. montevidensis* (1) |
| **Systemic lupus erythematosus (SLE)** | 1 | *Aspergillus terreus* |
| **Kaposi's sarcoma / HIV AIDS (4)** |  |  |
| **Kaposi's sarcoma (KS) / HIV AIDS** | 4 | *A. calidoustus* (1), *A. niger* (1), *A. sydowii* (1), *A. tubingensis* (1) |
